# Multi-modal integration of MRI and global chamber charge density mapping for the evaluation of atrial fibrillation

**DOI:** 10.1098/rsos.241048

**Published:** 2025-01-15

**Authors:** Alexander J. Sharp, Michael T. B. Pope, Andre Briosa e Gala, Richard Varini, Timothy R. Betts, Abhirup Banerjee

**Affiliations:** ^1^ Institute of Biomedical Engineering, Department of Engineering Science, University of Oxford, Oxford OX3 7DQ, UK; ^2^ Cardiology Department, John Radcliffe Hospital, Oxford University Hospitals NHS Foundation Trust, Oxford OX3 9DU, UK; ^3^ Cardiology Department, Southampton General Hospital, University Hospital Southampton NHS Foundation Trust, Southampton SO16 6YD, UK; ^4^ Division of Cardiovascular Medicine, Radcliffe Department of Medicine, University of Oxford, Oxford OX3 9DU, UK

**Keywords:** atrial fibrillation, atrial shape modelling, charge density mapping, magnetic resonance imaging, three-dimensional reconstruction

## Abstract

Atrial fibrillation (AF) is the most prevalent clinical arrhythmia, posing significant mortality and morbidity challenges. Outcomes of current catheter ablation treatment strategies are suboptimal, highlighting the need for innovative approaches. A major obstacle lies in the inability to comprehensively assess both structural and functional remodelling in AF. Combining magnetic resonance imaging (MRI)’s detailed structural insights with global chamber charge density mapping (CDM)’s functional mapping capabilities holds promise for advancing AF management. Our research introduces a novel tool for three-dimensional reconstruction of left atrial geometries from MRI, facilitating integration into CDM systems. We comprehensively assess our tool by generating three-dimensional left atrial meshes from MRIs of eight patients with AF and compare them with the established CDM intra-chamber ultrasound approach utilizing both geometric and clinical parameters. We apply the CDM inverse algorithm to both sets of reconstructions in order to compare derived conductions across various heart rhythms and AF conduction patterns. Finally, we explore the potential utility of our integrated pipeline through an exploration of the relationship between AF conduction patterns and their proximity to adjacent thoracic structures. Ultimately, this multifaceted approach aims to unveil insights into AF mechanisms, potentially improving treatment outcomes through personalized ablation strategies targeting arrhythmogenic atrial substrate.

## 1. Background

Atrial fibrillation (AF) is the most prevalent clinical arrhythmia globally and is associated with substantial morbidity and mortality. Current estimates indicate a prevalence ranging between 2 and 4% in adults, a figure anticipated to rise with improving recognition, diagnosis and an ageing population [1–3]. AF is characterized by chaotic electrical activity and propagation of depolarization within the atria, such that atrial contraction is ineffectual [4]. It requires both an initiating trigger and the presence of arrhythmogenic atrial substrate [5,6]. Negative atrial remodelling occurs during AF, and this promotes the formation of further arrhythmogenic atrial substrate. This concept of ‘AF begets AF’ is a key component in our understanding of its pathophysiology [7].

Since the landmark trial by Haïssaguerre *et al*. [8], which identified the significance of ectopic foci arising from the pulmonary veins as a trigger, pulmonary vein isolation (PVI) has formed the cornerstone of catheter ablation treatment for AF. However, particularly in patients with persistent AF (perAF), outcomes of this treatment remain suboptimal. Following a single catheter ablation procedure, AF recurs in approximately one-third of patients by 12 months. As such, patients frequently require multiple procedures, on average 1.5–1.7 [9,10]. Moreover, in 15–19% of these repeat procedures, it is found that the pulmonary veins are already successfully isolated [11,12], meaning that additional, non-pulmonary vein factors are responsible.

These observations have motivated efforts to develop catheter ablation approaches that additionally target arrhythmogenic atrial substrate through atrial substrate modification [10,13,14]. However, strategies of atrial substrate modification employing empirical isolation of atrial structures have not proven superior to PVI alone in randomized controlled trials [15–18]; this is likely due to the heterogeneity of this patient cohort, with a wide spectrum of atrial remodelling between individuals. This has led to the development of patient-specific approaches such as using global chamber charge density mapping (CDM).

While traditional contact mapping techniques are unable to globally map the rapidly changing activation patterns occurring in AF, CDM using the AcQMap system (Acutus Medical, CA, USA) enables visualization of conduction patterns in irregular rhythms [19–23]. This combined multi-electrode and imaging mapping system employs intra-chamber ultrasound (ICUS) to create a three-dimensional mesh of a patient’s atrial anatomy. Subsequently, an inverse algorithm is applied to non-contact intra-chamber voltage data to derive whole-chamber conduction in AF.

The hierarchical theory of AF defines a degree of organization within what was previously considered purely chaotic electrical activity. It describes discrete drivers of AF with underlying re-entrant, focal or rotational mechanisms; it is the propagation away from these drivers that is disorganized [5]. CDM can be used to guide atrial substrate modification by identifying potential AF drivers in the form of repetitive pathological conduction patterns (PCPs). These are divided into three categories: focal firing (FF), rotational propagation (localized rotational activation (LRA)), and pivoting propagation (localized irregular activity (LIA)) [19,22,23]. A complementary approach is the system’s ability to identify sites of consistent slow conduction velocity (CV) [24], with CV heterogeneity known to play a role in the initiation and maintenance of AF [25–27]. Results of a recent prospective, non-randomized study using the system have been encouraging, with a 76% freedom from AF at 12 months [23].

However, CDM is currently unable to assess the structural components of negative remodelling in AF. Cardiac magnetic resonance imaging (MRI) is a well-established method for investigating structural remodelling. It can accurately ascertain geometry, including wall thickness, and is the gold standard for quantifying atrial volumes [28,29]. Furthermore, it can characterize tissues, identifying areas of scar formation with late gadolinium enhancement [30,31]. Integration of MRI into CDM presents an exciting opportunity to elucidate the interplay between functional and structural remodelling in AF. It offers the potential to enhance our ability to identify successful ablation strategies beyond PVI through a comprehensive personalized approach for identifying areas of arrhythmogenic atrial substrate.

This study aims to develop a tool for the three-dimensional reconstruction of left atrial geometries from MRI, with a focus on its effective application to integrate MRI-derived geometries with CDM systems. By emphasizing this multi-model implementation, we address challenges that have not been explored in prior research and demonstrate significant potential for enhancing clinical workflows and decision-making. Specifically, our contributions are as follows:

— We generate three-dimensional left atrial reconstructions from eight patients with AF undergoing a catheter ablation procedure, using both our MRI-based approach and the current CDM ICUS approach, and perform co-registration.
— We compare MRI and ICUS reconstructions using both geometric and clinical metrics.
— We apply the CDM inverse algorithm to both MRI and ICUS reconstructions to compare derived conduction in terms of PCPs and CV.
— Utilizing our complete pipeline, we investigate the relationship between PCP locations within the left atria and their proximity to adjacent thoracic structures.

## 2. Methods

### 2.1. Study population

Eight patients with perAF, who had undergone an elective catheter ablation utilizing CDM, were retrospectively included in this study. This was a subset of a patient cohort recruited to a previous study (clinicaltrials.gov NCT04229472). Exclusion criteria included previous left atrial ablation, previous cardiac surgery and any contraindication to MRI scanning. All patients consented to their data being used in further studies. The investigation conformed to the principles outlined in the Declaration of Helsinki, and the original study protocol was approved by London–Surrey Research Ethics Committee (REC reference 20/LO/0150).

### 2.2. Study procedures

#### 2.2.1. Cardiovascular magnetic resonance imaging

One to 42 days before their scheduled ablation procedure, a cardiovascular MRI was performed using a 3 T scanner (MAGNETOM Prisma, Siemens Healthcare, Erlangen, Germany). Two and four chamber cine scans were acquired using a free-breathing, electrocardiographically gated sequence in sagittal and transverse orientations, respectively. Acquired voxel size was 2.8×2.8×8.0 mm^3^, repetition time 33.46 ms, echo time 1.05 ms and flip angle 50°.

#### 2.2.2. Charge density mapping-guided atrial fibrillation ablation

All procedures were performed under general anaesthetic. Venous access was obtained via bilateral femoral vein puncture under ultrasound guidance. Heparin boluses were administered prior to trans-septal puncture, followed by a continuous heparin infusion to maintain an activated coagulation time >350 s.

CDM using the AcQMap system has been previously discussed [20]. When deployed within the left atrium, the AcQMap catheter forms a 25 mm diameter spheroid consisting of six splines. Each spline contains eight ultrasound transducers and eight biopotential electrodes.

Geometry data were collected by rotating the catheter within the left atrium, enabling ultrasound point acquisition over the entire endocardial surface, the process usually taking 2–3 min. Intracardiac potentials were measured during both AF and sinus rhythm with atrial pacing. All measurements were made prior to any ablation. Mapping was completed in the presenting rhythm, followed by AF induction (using incremental burst atrial pacing) or termination (using external cardioversion) before completing the mapping procedures.

For measurements in AF, the AcQMap system was utilized in ‘single position’ mode. Here, the catheter is held stationary in the centre of the left atrium for the duration of the recording. A minimum of 60 s of AF was recorded. For measurements during sinus rhythm, the system’s ‘SuperMap’ high-resolution mapping mode was utilized [32]. During repetitive rhythms, this mode facilitates the accumulation of non-contact measurements taken at different times and locations within the left atrium. As such, the catheter can be manipulated around the atrium during a recording to bring it into closer proximity to the endocardium and increase the density of measurements. Mapping was undertaken during bipolar pacing from the mid-point of the coronary sinus catheter at a cycle length of 800 ms for four beats, followed by a single extra stimulus with a coupling interval of 300 ms (or the shortest captured cycle length if longer).

### 2.3. Three-dimensional left atrial endocardial surface mesh reconstruction

#### 2.3.1. From cardiovascular magnetic resonance

To enable integration of MRI reconstructions into CDM, the generated meshes need to be smooth and accurate representations of the left atrial endocardial surface [20]. We developed a custom, graphical user interface (GUI)-based tool implemented in MATLAB (The MathWorks, Natick, MA, USA) that enabled three-dimensional mesh reconstruction from two-dimensional cine MRI slices [33,34]. Our approach is fully automated following manual segmentation.

**Figure 1.**
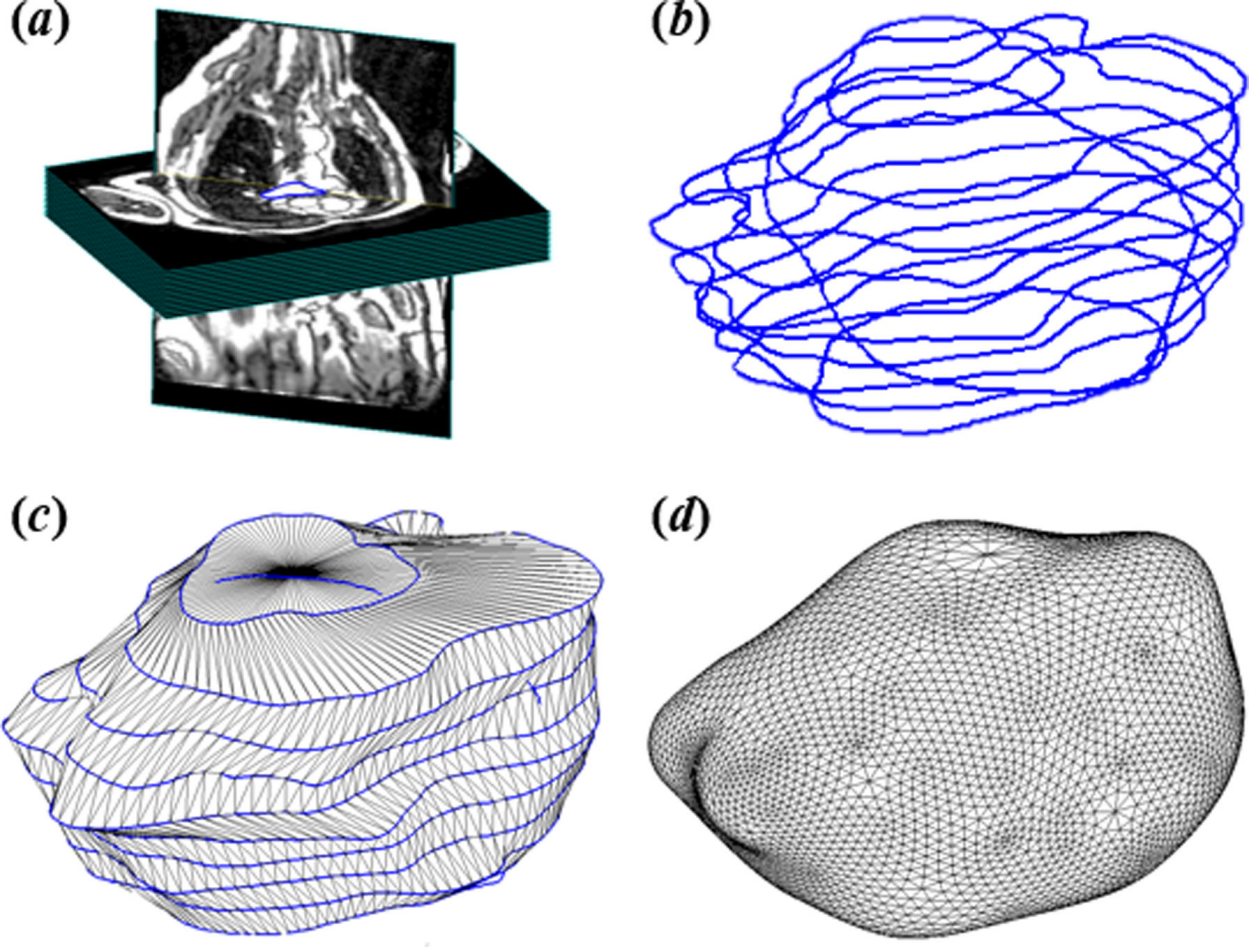
Left atrial endocardial surface mesh reconstruction from magnetic resonance imaging (MRI) contours. (*a*) Manual segmentation of MRI slices, (*b*) initial sparse representation by the alignment of segmented contours, (*c*) initial tubular three-dimensional mesh representation, (*d*) final three-dimensional left atrial shape following iterative optimization.

First, endocardial surfaces of the left atrium were manually segmented in the axial plane by an experienced clinician (figure 1*a*). The GUI facilitated the segmentation by aligning the slices into their true geometric locations with respect to the global coordinate system, i.e. the body surface. The additional segmentation of a mid-atrial transverse slice improved the alignment of axial slices. Following segmentation and extraction of the contours, the tool oriented them in their true three-dimensional locations to generate the initial sparse left atrium representation (figure 1*b*). From these sparse, heterogeneous, cross-sectional and non-coincidental contours, our approach automatically produced the three-dimensional left atrium surface mesh. First, we generated an initial three-dimensional representation of the left atrium as a tubular mesh by connecting the contours (figure 1*c*). Let $\left\{C_{j}\right\}$ be a set of contours lying on each two-dimensional MRI plane, with two additional points representing the lower and upper lid in order to generate a closed three-dimensional surface. By resampling K equally spaced vertices along the contours, we obtained the initial mesh using a ruled surface approach consisting of the initial triangles $\left\{\left(C_{j}\left(i\right),C_{j}\left(i+1\right),C_{j+1}\left(i\right)\right),\left(C_{j+1}\left(i\right),C_{j+1}\left(i+1\right),C_{j}\left(i+1\right)\right),\ldots \right\}$.

For generating the final three-dimensional mesh, we applied an attractor-based deformation step that iteratively minimized the distance of the mesh to the sparse contours representation while ensuring the smoothness and local topological properties [33]. Attractor points were defined along the contours using the farthest point sampling method [35], which iteratively pulled towards the closest points on the mesh in small consecutive steps, thus ensuring small, smooth deformations. Let *F* be the deformation field such that


$$arg\min_{F}\lambda \sum_{i}{\left(\left\|F\left(P_{i}\right)-Q_{i}^{\prime }\right\|\right)}^{2}+J_{2}^{3}\left(F\right)\;\;and\;\;Q_{i}^{\prime }=P_{i}+\beta \frac{Q_{i}-P_{i}}{\left\|Q_{i}-P_{i}\right\|},$$

where $\left\{P_{i}\right\}$ is the set of closest points on the surface $M_{t}$ to the attractor points, and $\left\{Q_{i}\right\}$ and $J_{2}^{3}$ are the thin plate splines functional using derivatives of order 2 and data dimensions 3. The deformation $M_{t+1}=F(M_{t})$ is applied in a diffeomorphic manner, with Laplacian smoothing, subdivision and decimation iteratively applied, resulting in the final three-dimensional left atrial shape (figure 1*d*). The Laplacian smoothing is performed as


$$L\left(p_{i}\right)=\frac{1}{|N_{i}|}\sum_{j\in N_{i}}p_{j}-p_{i},$$

where $p_{i}$ denotes the ith vertex and $p_{j}$ its adjacent vertices. This iterative optimization between attractor-based deformation towards the original contours and the Laplacian smoothing enabled us to create smooth reconstructions, which remained true to their segmentations, without the use of a template mesh [36] which may result in final reconstructions being biased towards the template.

#### 2.3.2. From intra-chamber ultrasound

Left atrial reconstructions from ICUS data were generated using IQ Viewer software (v. 3.7.10.150), which was developed in combination with the latest AcQMap 8.5 system (Acutus Medical, CA, USA). ICUS produces a point cloud representative of the left atrial endocardium. Prior to surface mesh reconstruction, points that lay significantly outside the main cloud were manually removed as per clinical practice, for example those due to reflections from the oesophagus. The surface mesh reconstructions from point clouds employed a global surface fitting approach to solving a three-dimensional Poisson equation. The Poisson approach incorporates a hierarchy of locally supported basis functions, reducing the solution to a well-conditioned sparse linear system. Accordingly, the approach is robust in the presence of significant variance in the point cloud and sensitive to structures in all orientations [37,38]. No smoothing was applied to the resultant reconstructions.

### 2.4. Registration and comparison of magnetic resonance imaging and intra-chamber ultrasound reconstructions

**Figure 2.**
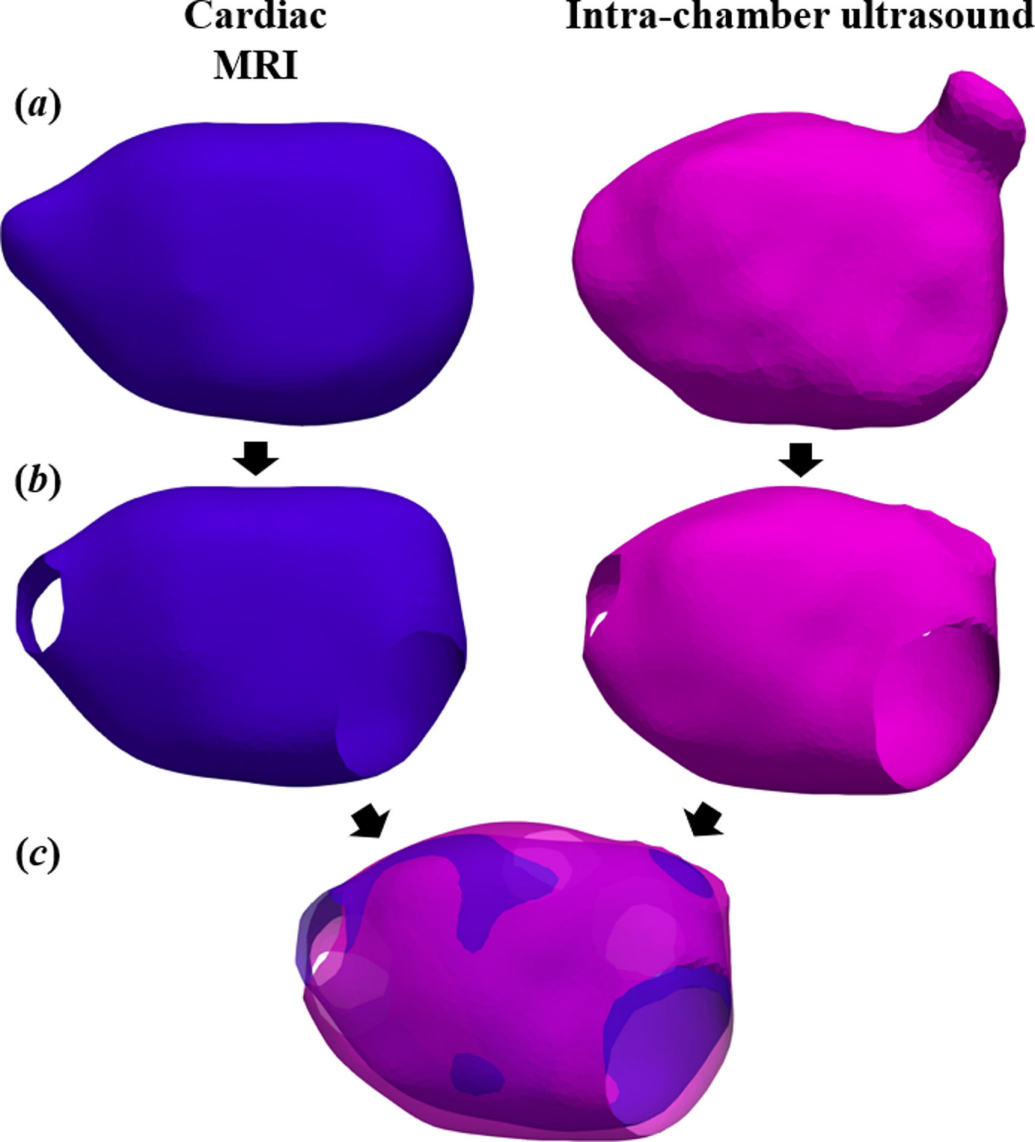
Registration of left atrial reconstructions. (*a*) Reconstructions from magnetic resonance imaging (MRI) contours and intra-chamber ultrasound (ICUS) point cloud; (*b*) removal of pulmonary veins, mitral valve, and left atrial appendage; (*c*) iterative closest point registration.

Individuals’ MRI and ICUS left atrial reconstructions (figure 1*a*) were registered using the generalized iterative closest point (ICP) algorithm [39] (figure 2*c*). The standard ICP algorithm calculates the optimum rigid transformation, i.e. translation and rotation, in order to minimize the distance between corresponding points on two geometries. Generalized ICP also considers the locally planar structure during iterative computation of the transformation, decreasing the influence of incorrect correspondences and improving the accuracy of registration.

Prior to registration, the mitral valve and pulmonary veins were excluded (figure 2*b*); these non-conductive structures are typically removed in the context of CDM [23]. Additionally, removal of the left atrial appendage, which is represented with limited accuracy in ICUS reconstructions, improved registration. This limited modification of the surface meshes was performed using ACMFrame software (Acutus Medical, CA, USA). Registration performance was first evaluated qualitatively through visualization of three-dimensional co-registered geometries. Quantitative comparison was performed through geometric evaluation, using surface distance between registered reconstructions, as well as through clinical evaluation metrics, using left atrial volume.

### 2.5. Comparison of derived whole-chamber conduction

Following registration, individuals’ MRI and ICUS left atrial reconstructions were imported into ACMFrame software. Both reconstructions were used to derive conduction from the intracardiac potentials recorded during their ablation procedures.

Each reconstruction consists of approximately 3000 vertices. Following analysis of all mapping protocols, each vertex received a measurement for:

— CV during sinus rhythm with long cycle length pacing;
— CV during sinus rhythm with short cycle length pacing;
— median CV during a 20 s segment of AF; and
— frequency of PCPs, which may represent AF drivers: FF, LRA, LIA.

Differences in derived conduction between MRI and ICUS reconstructions were assessed at an individual level through histograms of CV and PCP frequency, against surface area of reconstructions. To enable comparison at a population level, values at each vertex of individuals’ MRI reconstructions were assigned to the corresponding vertex of their co-registered ICUS reconstructions using the nearest neighbour method. Subsequently, population-wide relationships were assessed through two-dimensional histograms for CV and contingency heatmaps for PCP values.

### 2.6. Combining charge density mapping and magnetic resonance imaging reconstructions of adjacent thoracic structures

**Figure 3.**
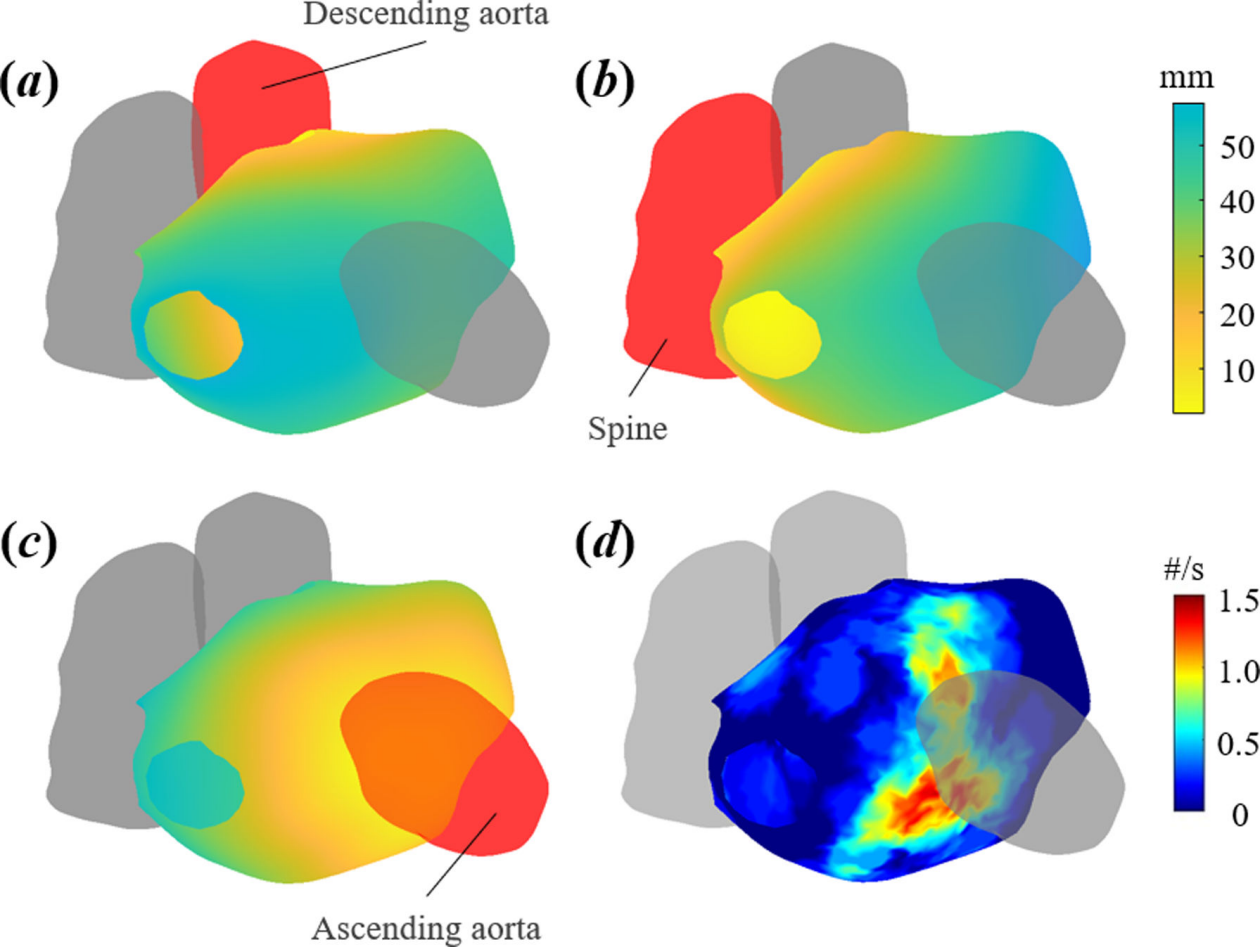
Combining charge density mapping and magnetic resonance imaging reconstructions of the left atrium, and its adjacent thoracic structures (in red). (*a–c*) Euclidean distance between left atrium reconstruction and descending aorta (*a*), spine (*b*) and ascending aorta (*c*). (*d*) Frequency of localized rotational activation in the context of adjacent structures.

Previous studies have suggested that the left atrium adjacent thoracic structures of the ascending aorta, descending aorta and spine may be implicated in negative atrial remodelling [40–43]. Using our developed GUI-enabled three-dimensional reconstruction tool, these structures were first segmented in the axial plane of individuals’ MRI scans, and accurate three-dimensional reconstructions automatically generated as described in detail in §2.3.1. The same transformation that registered individuals’ MRI and ICUS left atrial reconstructions was then applied to these adjacent structures, enabling left atrial reconstructions with their associated PCP frequencies to be analysed in the context of these adjacent structures (figure 3*d*). The Euclidean distance between each vertex of left atrial reconstructions and the closest point on these adjacent structures was calculated (figure 3*a*–*c*).

### 2.7. Statistical analysis

Continuous data are described as mean ± standard deviation (s.d.) if normally distributed or as median (first quartile (Q1); third quartile (Q3)). Categorical data are described with absolute and relative frequencies.

Statistical analysis was performed using R statistical software (v. 4.3.1) and RStudio (v. 2023.09.1). The paired Student’s *t*‐test was used in comparing volumes of MRI and ICUS reconstructions. Correlation between derived CVs and PCP frequencies was assessed using Pearson correlation coefficient and Spearman correlation, respectively. The Mann–Whitney *U* test was used in comparing the frequency of PCPs and proximity to adjacent thoracic structures. All statistical tests were two sided; *p* < 0.05 was considered statistically significant.

## 3. Results

### 3.1. Patient and imaging characteristics

The clinical characteristics of all patients are outlined in table 1. Antiarrhythmic medications (except amiodarone) were stopped a minimum of 5 days before catheter ablation procedures.

**Table 1.** Clinical characteristics of the study population.

characteristic	patient cohort (*n* = 8)
age, years, mean ± s.d.	64.6 ± 9.4
sex	
men	4 (50%)
women	4 (50%)
BMI, kg m^−2^, mean ± s.d.	31.5 ± 4.6
CHA2DS2-VASc score, *n* (%)	
0	1 (13%)
1	2 (25%)
2	1 (13%)
3	3 (38%)
4	1 (13%)
time from first diagnosed AF, years, median (IQR)	1 (1–3.75)
ablation type, *n* (%)	
de novo	5 (63%)
retreatment	3 (38%)

BMI, body mass index; IQR, interquartile range; s.d., standard deviation.

Characteristics of both MRI and ICUS imaging are outlined in table 2. Mean duration of ICUS recording was 132±15 s. Median time between MRI and ablation procedure was 3 days. Four (50%) patients were in a different rhythm during their MRI and ICUS imaging.

**Table 2.** Characteristics of MRI and ICUS imaging.

case	rhythm during MRI	rhythm during ICUS	duration ICUS collection (min:s)	time between MRI and ICUS (days)
1	AF	AF	2:21	17
2	AF	SR	2:21	3
3	AF	SR	2:05	3
4	AF	AF	1:50	3
5	SR	AF	2:36	3
6	SR	SR	2:12	3
7	SR	AF	2:12	3
8	AF	AF	1:56	10

AF, atrial fibrillation; ICUS, intra-chamber ultrasound; MRI, magnetic resonance imaging; SR, sinus rhythm.

### 3.2. Performance of magnetic resonance imaging reconstructions

Reconstruction performance was evaluated by the distance between contours and the reconstructed three-dimensional surface for each case (table 3). Average distance between MRI contours and reconstructed left atrial geometry was 1.4±1.2 mm, which is markedly below the underlying resolution of the acquired MRI slices (voxel size of 2.8 × 2.8 × 8.0 mm^3^).

**Table 3.** Distance between MRI contours and reconstructed geometry for each case.

case	distance, mean ± s.d. (mm)
1	1.4 ± 1.1
2	1.0 ± 1.0
3	1.5 ± 1.1
4	1.8 ± 1.6
5	1.3 ± 1.1
6	1.2 ± 1.2
7	1.2 ± 1.1
8	1.3 ± 1.5

MRI, magnetic resonance imaging; s.d., standard deviation.

### 3.3. Comparison of magnetic resonance imaging and intra-chamber ultrasound reconstructions

Mean distances between individual patients’ registered reconstructions are shown in table 4. Average of the mean distance between reconstructions was 1.5±2.0 mm, which is reasonable considering the MRI voxel size (2.8 × 2.8 × 8.0 mm^3^). Reconstructions produced from ICUS had a significantly larger left atrial volume than the corresponding MRI reconstructions (table 4): 166.9±23.5 ml from ICUS versus 144.2±21.2 ml from MRI (*p* < 0.001). Removing Laplacian smoothing from our MRI reconstruction tool had no significant impact on volume prior to mitral valve, pulmonary veins and left atrial appendage exclusion: 148.9±20.4 ml with smoothing versus 151.0±21.7 ml without smoothing (*p* = 0.09).

**Table 4.** Mean distances between, and left atrial volumes of, MRI and ICUS reconstructions.

case	distance between reconstructions, mean ± s.d. (mm)	volume from ICUS (ml)	volume from MRI (ml)	relative volume of MRI versus ICUS (%)
1	1.6±2.3	184.8	154.0	83.4
2	1.4±1.7	171.7	147.9	86.2
3	1.4±1.7	145.6	125.0	85.8
4	1.5±2.8	191.3	163.2	85.3
5	1.4±2.8	165.4	136.7	82.7
6	1.7±1.8	142.1	117.4	82.6
7	1.6±1.8	197.8	180.2	91.1
8	1.4±1.5	136.8	129.2	94.4

ICUS, intra-chamber ultrasound; MRI, magnetic resonance imaging; s.d., standard deviation.

### 3.4. Inverse solution-derived whole-chamber conduction

**Figure 4.**
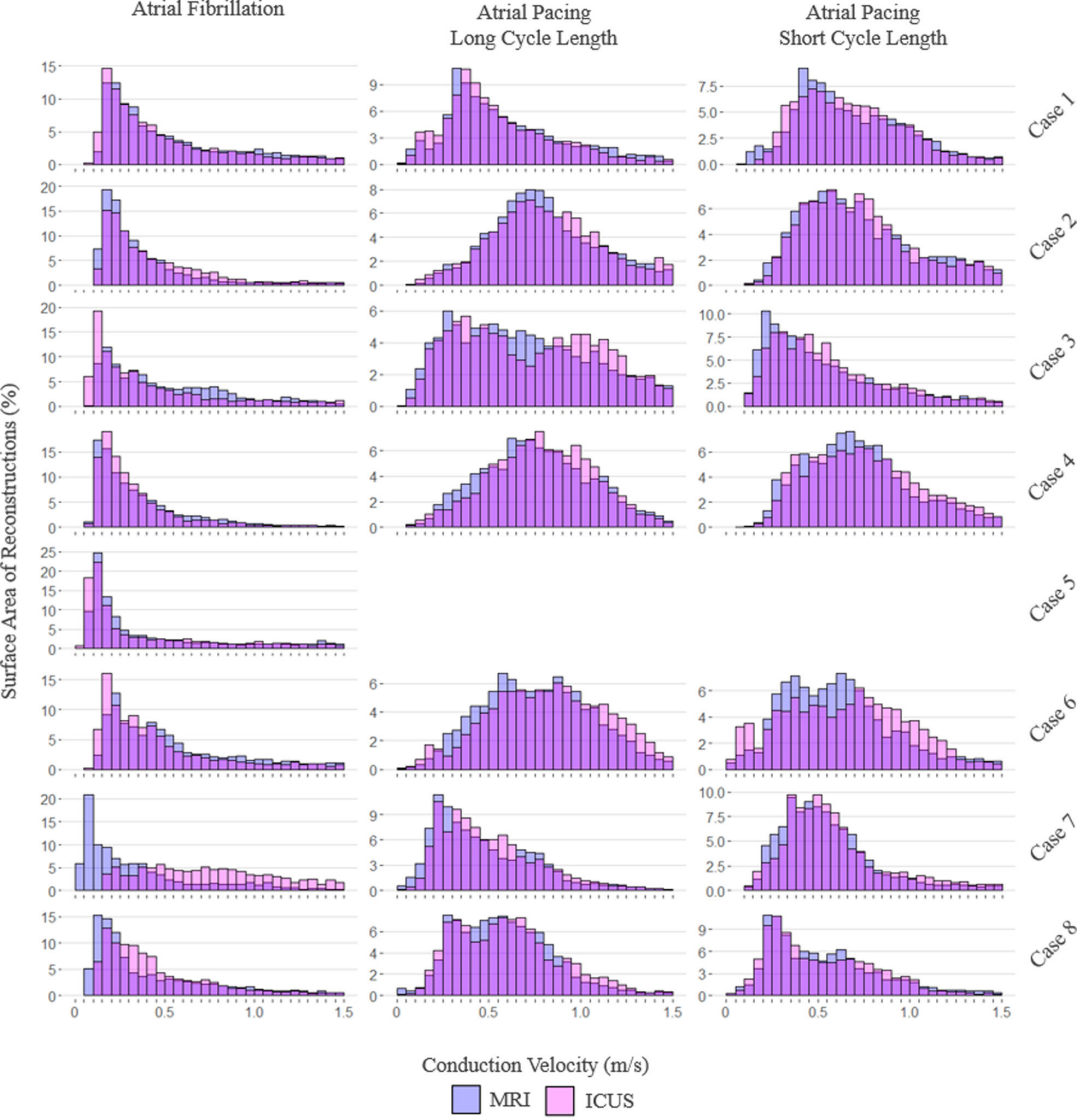
Histograms of conduction velocity versus surface area of magnetic resonance imaging (MRI) and intra-chamber ultrasound (ICUS) reconstructions, dependent on atrial rhythm.

**Figure 5.**
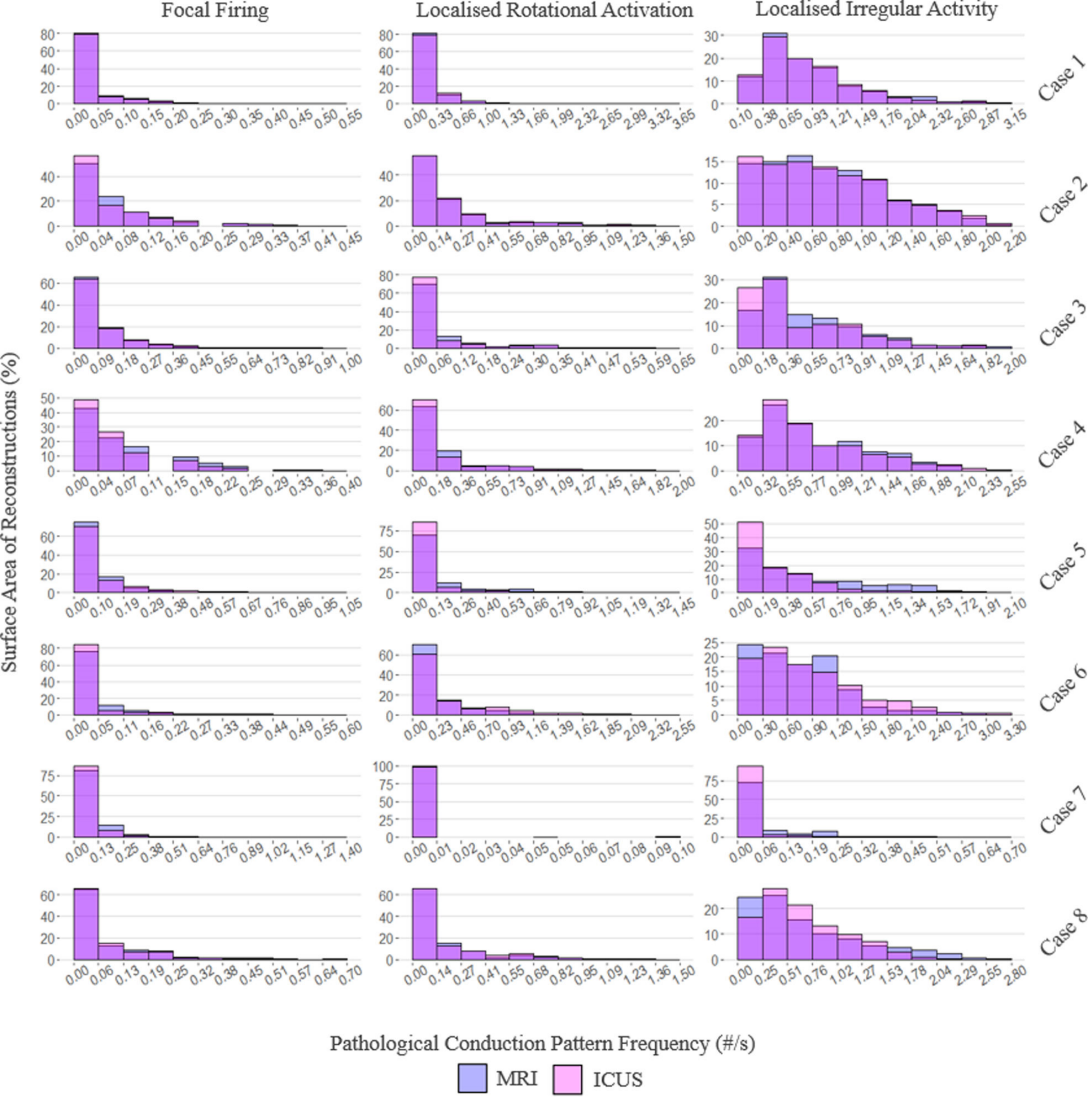
Histograms of pathological conduction pattern frequencies versus surface area of magnetic resonance imaging (MRI) and intra-chamber ultrasound (ICUS) reconstructions.

Histograms of CV versus surface mesh area utilizing MRI and ICUS reconstructions are presented in figure 4, with a CV threshold of 1.5 m s^−1^ applied in line with physiological relevance. PCP frequencies versus surface mesh area are presented in figure 5. Variable *x*- and *y*-axis scales are used due to the variable distributions of these measures between cases. In one instance (case 5), it was not possible to maintain sinus rhythm for recordings of CV during atrial pacing.

**Figure 6.**
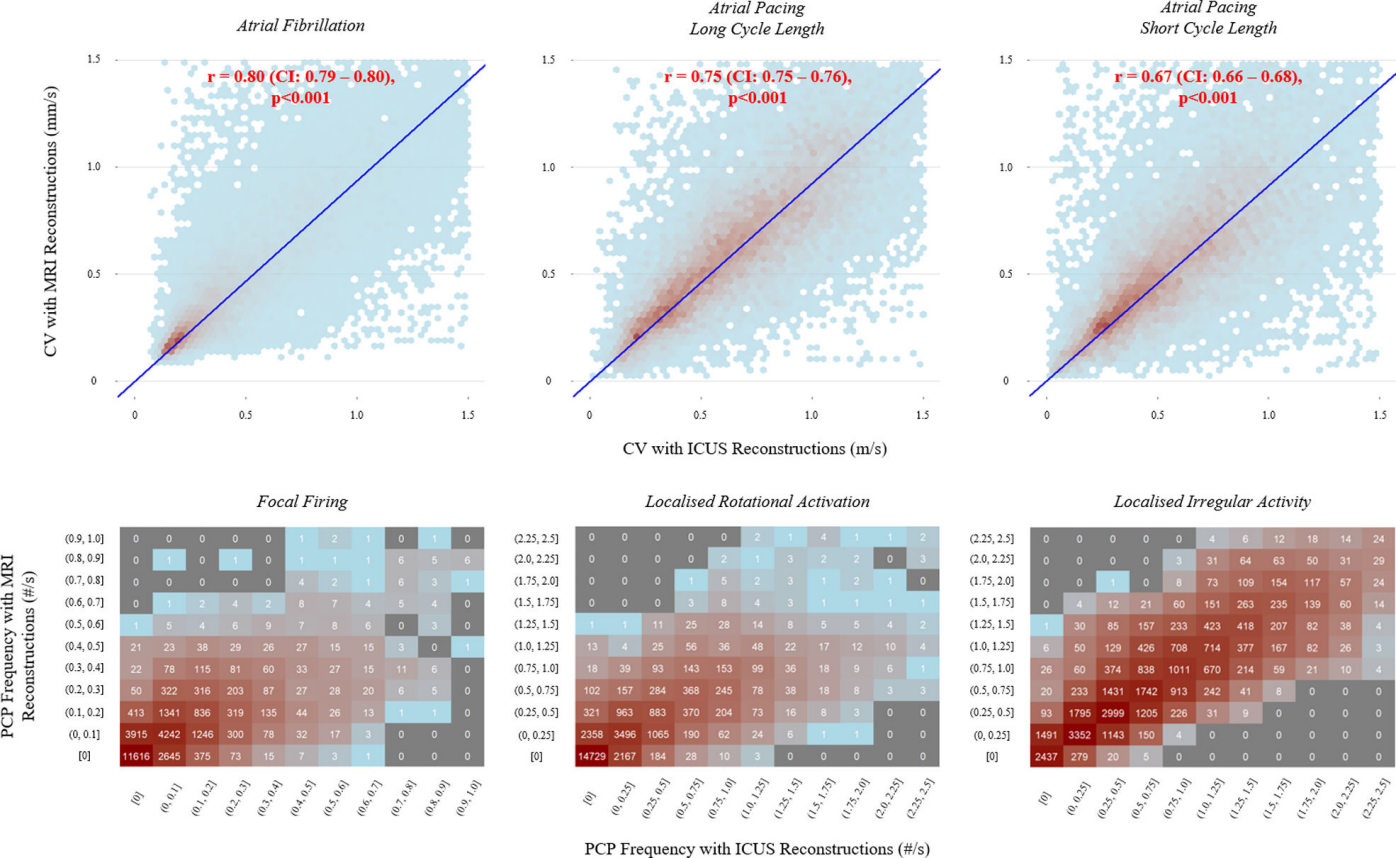
Population-wide relationships dependent on reconstruction modality. Top: two-dimensional histogram (hexbin) plots with linear regression lines (blue) of conduction velocity; bottom: contingency heatmaps of conduction pattern frequencies. CV, conduction velocity; ICUS, intra-chamber ultrasound; MRI, magnetic resonance imaging; PCP, pathological conduction patterns.

At a population level, there were strong to moderate linear relationships between MRI and ICUS reconstruction-derived conduction across all measures (figure 6). The correlations between CV were as follows: during AF, *r* = 0.80 (confidence interval (CI): 0.79–0.80, *p* < 0.001); during long cycle length atrial pacing, *r* = 0.75 (CI: 0.75–0.76, *p* < 0.001); and during short cycle length atrial pacing, *r* = 0.67 (CI: 0.66–0.68, *p* < 0.001). The correlations between the PCP frequencies were as follows: FF, *r* = 0.60, *p* < 0.001; LRA, *r* = 0.73, *p* < 0.001; and LIA, *r* = 0.89, *p* < 0.001.

### 3.5. Impact of adjacent thoracic structures

**Figure 7.**
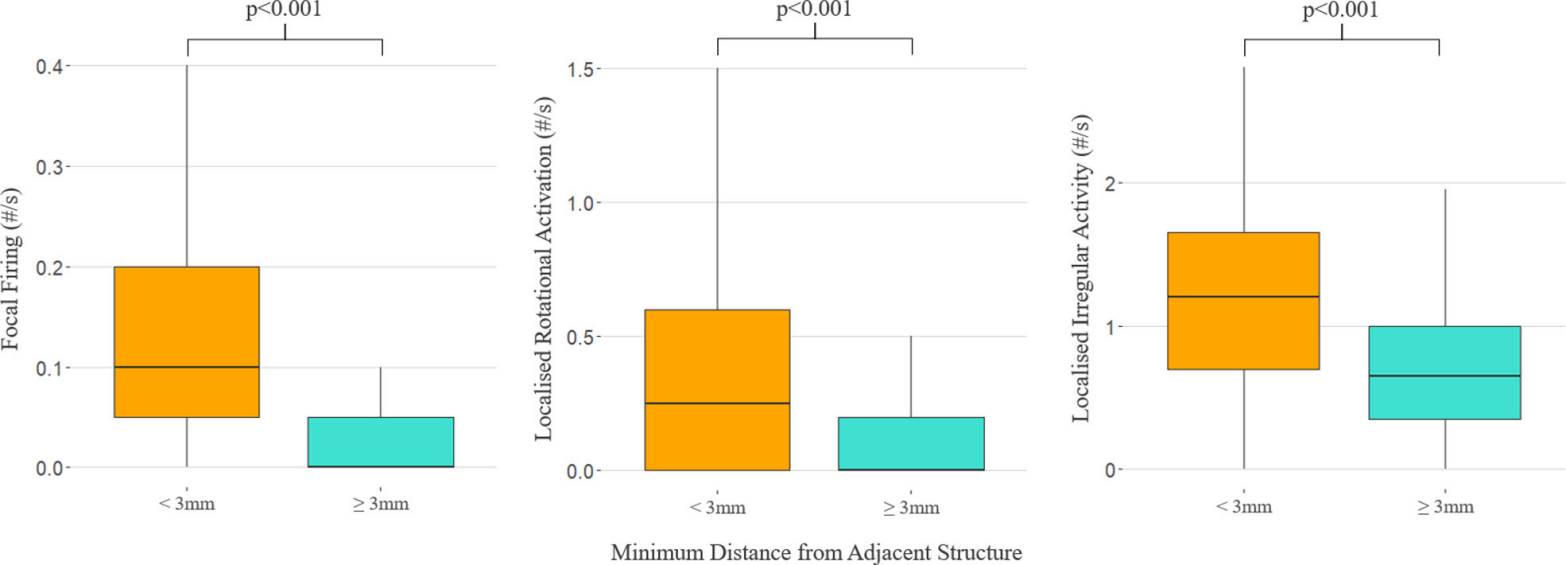
Frequencies of pathological conduction patterns, depending on the proximity of vertices of left atrial reconstructions to adjacent thoracic structures.

Consistent with previous literature [43,44], we considered any vertices of left atrial reconstructions within 3 mm of an adjacent thoracic structure to be in contact. Frequencies of all PCP categories were significantly higher at these vertices (figure 7): median FF 0.1 s^−1^ (interquartile range (IQR) 0.05–0.2) versus 0 s^−1^ (IQR 0–0.05), *p* < 0.001; median LRA 0.25 s^−1^ (IQR 0–0.6) versus 0 s^−1^ (IQR 0–0.2), *p* < 0.001; and median LIA 1.2 s^−1^ (IQR 0.7–1.65) versus 0.65 s^−1^ (IQR 0.35–1), *p* < 0.001.

## 4. Discussion

To the best of our knowledge, this is the first study that has utilized MRI-derived left atrial reconstructions in CDM of AF.

### 4.1. Left atrial reconstructions

Our tool produced accurate and smooth left atrial reconstructions from MRI, with average misalignment of reconstructions to MRI contours (1.4±1.2 mm) comparing favourably to the in-plane resolution (2.8×2.8 mm^2^).

When compared to the current clinical standard of ICUS, our average distance between reconstructions of 1.5±2.0 mm demonstrates performance on par with previous work utilizing computed tomography in a nine-patient cohort where the absolute median distance between surfaces was 1.85 mm [20]. Additionally, this similarity is demonstrated using the updated reconstruction techniques from ICUS implemented into AcQMap 8.5 and IQViewer software; these promise improved accuracy, utilizing a novel application of a Poisson-based method, rather than the previous radial binning approach, which locally averaged distances within a set of polar angles and suffered from fidelity loss when reconstructing radially oriented structures [37]. Of note, our MRI and ICUS reconstructions were similar even in cases where imaging was performed in differing rhythms, thus improving the clinical applicability of our methodology.

Our MRI reconstructions were significantly smaller in volume than those from ICUS. In accounting for this observation, both clinical and technical explanations need to be considered. Clinically, we may have expected smaller ICUS volumes, with this being performed with patients in a fasted state for general anaesthetic and prior to any irrigated ablation, which may have influenced volume loading [45]. However, general anaesthetic has been observed to increase the size of atria, likely through a negative inotropic effect leading to atrial dilatation [46].

**Figure 8.**
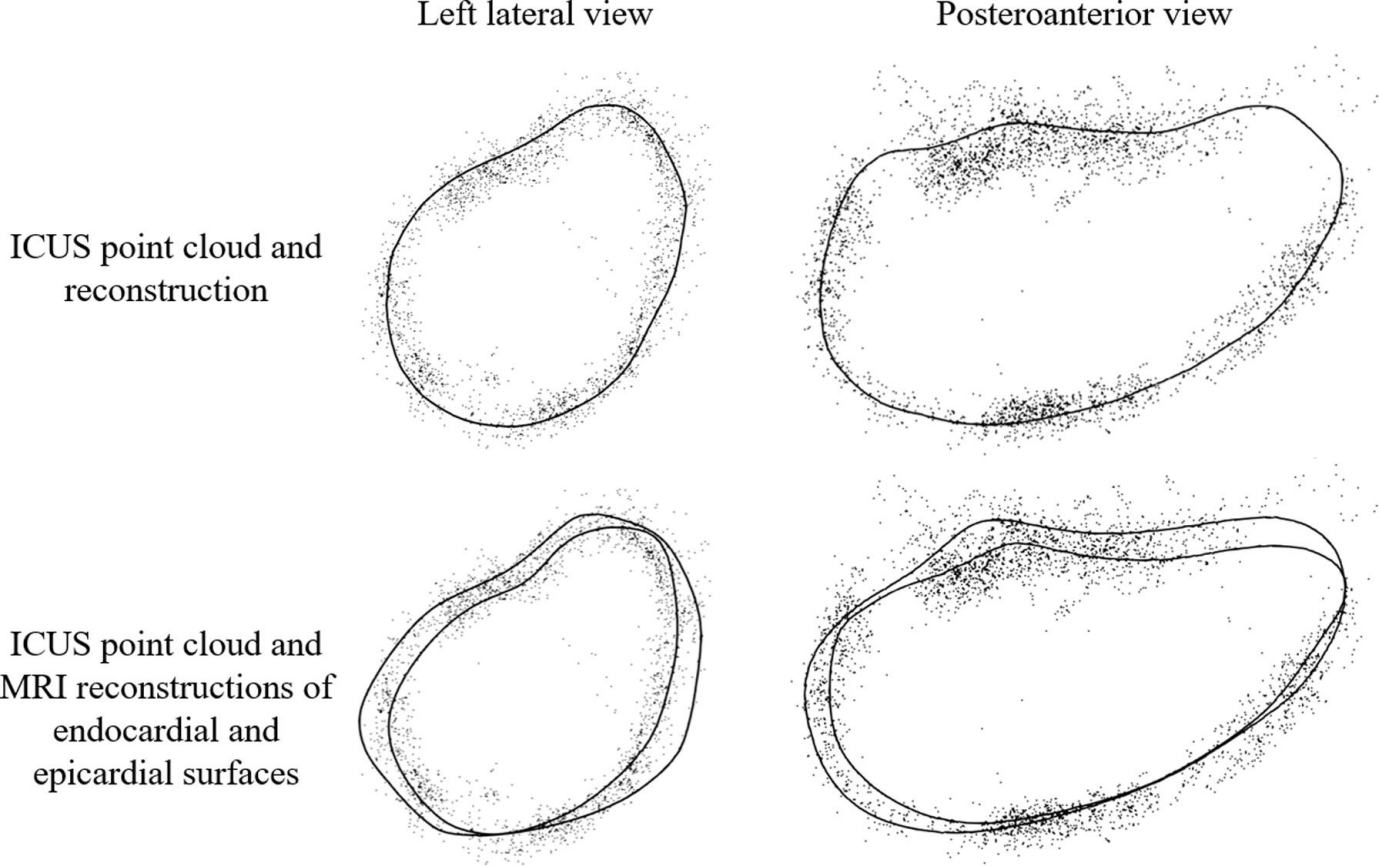
Example (case 6) of intra-chamber ultrasound (ICUS) point cloud in combination with left atrial reconstruction from ICUS (top) and endocardial and epicardial reconstructions from MRI (bottom). ICUS point cloud may contain both epicardial and endocardial reflections from the left atrium, with consequentially larger reconstructions.

Technically, the inclusion of Laplacian smoothing within our MRI reconstruction tool accounted for a minimal decrease in volume; however, this difference was not observed to be significant compared to reconstructions without Laplacian smoothing. Instead, we hypothesized that ICUS point clouds contained both epicardial and endocardial reflections from the left atrium, with consequentially larger reconstructions. Visualization of ICUS reconstructions in combination with their respective point clouds and comparing this to MRI-based endocardial and epicardial reconstructions supported this hypothesis (figure 8). Quantitatively, we calculated the difference in volume between MRI endocardial wall and ICUS reconstructions and divided this by the surface area of MRI endocardial wall reconstructions to calculate the equivalent additional wall thickness. Across our cohort, this was 1.9±0.63 mm providing further support for our hypothesis, previous anatomical studies of the left atrium finding a mean wall thickness between 2.3 and 4.5 mm [47]. As such, our MRI reconstructions are likely more accurate representations of the left atrial endocardium.

### 4.2. Derived conduction

MRI reconstructions using our tool were successfully integrated into CDM to derive conduction from non-contact intra-chamber voltage measurements.

Cycle length is known to impact CV [27,48]. As such, our comprehensive assessment considered a range of different protocols, with measurements being taken during AF and sinus rhythm with both long and short cycle length pacing. At an individual level, visual inspection of histograms revealed similar results for CV distributions between reconstructions. Cases 6 and 7 displayed the most discrepancy during AF and short cycle length atrial pacing, respectively. These cases had the largest mean distances between ICUS and MRI reconstructions, which likely accounts for this. Interestingly, case 7 had the second-best concordance in left atrial volume, implying that at these high levels of geometric similarity, accurate shape may be a more important factor than scale in deriving CV. At a population level, correlation was strongest during AF. CDM’s strength is in globally mapping such irregular rhythms; as such, this result is highly encouraging given the intended clinical utility of our tool.

Regarding PCPs, visual inspection of individual histograms revealed similarly good results. Both FF and LRA histograms displayed a strong positively skewed distribution; the lower frequencies of these PCPs likely contributed to the weaker correlations observed at a population level. These lower frequencies are in line with a previous study of 103 patients, which reported relative frequencies of PCPs as: LIA 74.4%, LRA 14.5% and FF 11.1% [23]. This observed higher frequency of LIA, in combination with strong concordance between reconstruction modalities in its measurement, is again encouraging in the suitability of our tool clinically for assisting in the identification of the most prevalent AF drivers.

### 4.3. Impact of adjacent thoracic structures

Our work is the first to use CDM to establish an association of PCPs representing possible AF drivers with proximity to adjacent left atrial structures. This is mechanistically plausible, with compression from adjacent structures a source of atrial wall deformation and wall stress that could contribute to negative atrial remodelling [41,49]. Mechanical stress is a key regulator of cardiac electrophysiology, particularly within the low-pressure atria, where mechanosensors in the form of stretch-activated ion channels are known to exist [50,51]. Indeed, many risk factors in the development of AF (age, hypertension, obesity, obstructive sleep apnoea, etc*.*) are united mechanistically, through elevated atrial pressure and wall stress [52,53].

Our results are in line with previous studies demonstrating an association between adjacent structures and remodelling identified as low-voltage areas, fractionated electrograms and late gadolinium enhancement [40,42,43], with the advantage that CDM allows us to observe and quantify conduction patterns indicative of AF drivers, rather than relying on surrogate measures of remodelling. Our observations support the hypothesis that negative atrial remodelling may be influenced by extrinsic anatomical features and open avenues for further studies exploring whether this observation represents causation rather than correlation and if the location of these structures should be considered in the planning and execution of ablation procedures.

### 4.4. Clinical significance

The integration of CDM with MRI-derived structural information provides unique insights into both the electrical and tissue-level characteristics of the left atrium. This could potentially lead to a more tailored approach to AF treatment, where ablation strategies are based not only on electrical propagation patterns but also on tissue properties such as fibrosis and extracellular volume, which are important contributors to arrhythmogenic substrate.

We recognize that the clinical application of such an integrated approach will require further validation and refinement. While this study is not intended to provide a definitive clinical solution, it serves as a critical first step in demonstrating the feasibility of integrating these data types to better understand the complex pathophysiological relationships responsible for AF. By demonstrating that the combination of these modalities is feasible and potentially beneficial, we lay the groundwork for future studies that could eventually lead to new, more personalized ablation strategies with improved clinical outcomes.

### 4.5. Study limitations

While our study provides valuable insights into the integration of MRI and CDM in the mapping and treatment of AF, it included a limited number of patients, which may affect the generalizability of our findings. CDM-derived electrograms have been validated against contact mapping in both SR and AF [54], and the benefit of targeting abnormal propagation patterns is demonstrated in single-arm cohort studies [21–23]; however, randomized controlled trials are lacking. Our current investigation focused on the left atrium since it is the most frequently targeted; however, our proposed methodology could be applied to bi-atrial assessments. Integration of MRI into CDM continues to pose technical challenges, and the approach presented here describes important complementary roles of both modalities.

## 5. Conclusions

We have successfully developed a novel tool for three-dimensional reconstruction of left atrial geometries from MRI, which can be integrated with CDM systems to provide both structural and functional insights into left atrial remodelling in AF. Our work provides the foundation for further integration of MRI’s ability to characterize tissues and personalizing AF therapy through improved understanding of an individual’s arrhythmogenic atrial substate. Consequently, clinicians may deliver optimized ablation strategies that improve outcomes in that individual and reduce the need for repeat procedures.

## Data Availability

All reconstructed three-dimensional meshes from MRI and ICUS imaging modalities, and the processing codes implemented in R, are provided at Figshare [55].
